# Respiratory Syncytial Virus Promotes *Moraxella catarrhalis*-Induced Ascending Experimental Otitis Media

**DOI:** 10.1371/journal.pone.0040088

**Published:** 2012-06-29

**Authors:** M. Elizabeth Brockson, Laura A. Novotny, Joseph A. Jurcisek, Glen McGillivary, Martha R. Bowers, Lauren O. Bakaletz

**Affiliations:** The Research Institute at Nationwide Children’s Hospital, Center for Microbial Pathogenesis and The Ohio State University College of Medicine, Columbus, Ohio, United States of America; Lovelace Respiratory Research Institute, United States of America

## Abstract

Otitis media (OM) is a polymicrobial disease wherein prior or concurrent infection with an upper respiratory tract virus plays an essential role, predisposing the middle ear to bacterial invasion. In episodes of acute bacterial OM, respiratory syncytial virus (RSV) is the most commonly isolated virus and thus serves as an important co-pathogen. Of the predominant bacterial agents of OM, the pathogenesis of disease due to *Moraxella catarrhalis* is the least well understood. Rigorous study of *M.* *catarrhalis* in the context of OM has been significantly hindered by lack of an animal model. To bridge this gap, we assessed whether co-infection of chinchillas with *M. catarrhalis* and RSV would facilitate ascension of *M. catarrhalis* from the nasopharynx into the middle ear. Chinchillas were challenged intranasally with *M. catarrhalis* followed 48 hours later by intranasal challenge with RSV. Within 7 days, 100% of nasopharynges were colonized with *M. catarrhalis* and homogenates of middle ear mucosa were also culture-positive. Moreover, within the middle ear space, the mucosa exhibited hemorrhagic foci, and a small volume of serosanguinous effusion was present in one of six ears. To improve upon this model, and based on epidemiologic data, nontypeable *Haemophilus influenzae* (NTHI) was included as an additional bacterial co-pathogen via intranasal administration four days before *M. catarrhalis* challenge. With this latter protocol, *M. catarrhalis* was cultured from the nasopharynx and middle ear homogenates of a maximum of 88% and 79% animals, respectively, for up to 17 days after intranasal challenge with *M. catarrhalis*. Additionally, hemorrhagic foci were observed in 79% of middle ears upon sacrifice. Thus, these data demonstrated that co-infection with RSV and NTHI predisposed to *M. catarrhalis*-induced ascending experimental OM. This model can be used both in studies of pathogenesis as well as to investigate strategies to prevent or treat OM due to *M. catarrhalis*.

## Introduction

Otitis media (OM), inflammation of the middle ear, is a highly prevalent pediatric disease globally [1], [2]. Unlike many other infectious diseases, bacterial OM is not caused by classically virulent microorganisms, but rather by commensals of the pediatric nasopharynx (NP) that can nonetheless behave as opportunistic pathogens when conditions are optimal for them to do so. OM is a significant cause of morbidity and has many important sequelae, such as conductive hearing loss [3], [4] accompanied by delays in language, behavioral and cognitive development [2], [5]. In addition, the direct and indirect costs associated with OM are estimated to exceed $5 billion annually in the United States alone [1], [6], [7], [8].

OM is often associated with preceding or concurrent viral upper respiratory tract infection, such as those caused by parainfluenza virus [9], influenza virus [10], [11], [12], rhinovirus [13], adenovirus [14] and respiratory syncytial virus (RSV) [9], [13]. Infection by upper respiratory tract viruses results in dysregulation of normal Eustachian tube (ET) function via decreased mucociliary action, altered mucus secretion by goblet cells and increased expression of inflammatory mediators of the host [15], [16], [17], among other mechanisms. The resultant transient reduction in protective functions of the ET provides the opportunity for commensal bacteria of the NP to ascend into the middle ear and cause disease. The three most common bacterial causative agents of OM are *Streptococcus pneumoniae*, nontypeable *Haemophilus influenzae* (NTHI) and *Moraxella catarrhalis*, all of which are normal commensal species of the pediatric NP [18], [19], [20]. To better understand the pathogenesis of OM, animal models are utilized in the study of all three predominant pathogens of OM [21], [22], [23], [24], [25], [26], [27], [28], [29], [30], [31], [32]; however, the lack of a model wherein *M. catarrhalis* ascends into the middle ear has strongly hindered studies of *M. catarrhalis*-induced OM.

Whereas there are multiple rodent models of experimental OM caused by bacteria, the majority of these require direct inoculation of bacteria into the middle ear space [20], [22], [33], [34]. To date, animal models of OM that demonstrate bacterial ascension from the NP into the middle ear space exist only for *S. pneumoniae* [33] and NTHI [22], not *M. catarrhalis*. This is despite the fact that *M.* *catarrhalis* is an increasingly important OM pathogen, particularly after the widespread use of the heptavalent pneumococcal conjugate vaccine [35], [36], [37], [38], and nasopharyngeal carriage of this microorganism is more prevalent in some sub-populations, such as Australian Aborigines [19], [39], [40]. Recent studies of Aboriginal children with acute OM (AOM) demonstrate that at least 95% of nasopharyngeal swabs were positive for *M. catarrhalis* by either culture [40] or quantitative real time PCR [19]. Nonetheless, little is known about the pathogenesis of *M.* *catarrhalis*-induced OM. The ability to better model this pediatric disease experimentally, and in a way that more faithfully replicates the natural disease course, would likely allow for a greater understanding of *M. catarrhalis* pathogenesis.

Given that viral infection of the upper respiratory tract predisposes children to bacterial OM, established animal models of bacterial ascension of the ET incorporate a viral partner to induce bacterial OM [23], [24], [41], [42], [43], [44], [45], [46]. As RSV is a predominant viral co-pathogen of OM [19], [47], and because other viral partners such as adenovirus have failed to predispose to *M. catarrhalis*-induced OM in an animal model [48], we hypothesized that RSV might serve as an appropriate viral partner for *M. catarrhalis*. Moreover, epidemiologic studies have demonstrated a high incidence of *M. catarrhalis* co-colonizing the nasopharynx of children with another bacterial species [19], [49], [50], and one study demonstrated that when *M. catarrhalis* is cultured from the middle ear of children with AOM, it is found with at least one other bacterial species in 67% (331 of 496) of cases [51]. In 66% (218 of 331) of those polymicrobial cases, *M. catarrhalis* is co-cultured with *H. influenzae*. In addition to compelling epidemiologic data, Armbruster *et al*. have demonstrated in an experimental transbullar challenge model that the presence of NTHI increases the persistence of *M. catarrhalis* in the chinchilla middle ear [25]. We therefore further hypothesized that *M. catarrhalis* might require a bacterial co-pathogen in addition to a viral co-pathogen in order to induce ascending OM.

It is established that juvenile chinchillas are permissive to infection with RSV. Work by Gitban *et al.* demonstrates signs of upper respiratory tract infection, including compromise of ET function, goblet cell hyperplasia and increased mucus production within four days after intranasal challenge with RSV [44], and maximal virus-induced middle ear underpressure only two days after challenge. Moreover, virus-neutralizing antibody is detected within the serum and indicates that chinchillas respond immunologically to experimental infection with RSV. Grieves *et al.* utilized a red fluorescent protein-expressing RSV to examine the kinetics of viral infection after intranasal challenge of juvenile chinchillas and observed that within 5 days after challenge, the RSV fluorescent signal advanced from the site of inoculation, through the nasopharynx and throughout the ET [52]. Thus, experimental infection with RSV, and likely also virus-induced alterations to this respiratory epithelium, extended throughout the uppermost respiratory tract.

Building upon these data, we partnered RSV with *M. catarrhalis* to identify whether changes induced by prior RSV infection would facilitate ascension of *M. catarrhalis* from the nasopharynx into the middle ear. We first performed a study wherein 24, 36, or 96 hours after viral challenge (time points which encompassed the period of maximal RSV-induced ET dysfunction in an attempt to promote the opportunity for *M. catarrhalis* to gain access to the middle ear), juvenile chinchillas were challenged with *M. catarrhalis*. Disappointingly, few, if any signs of middle ear disease were observed. To refine this protocol, and based on the previously described epidemiologic data in which NTHI is co-cultured from clinical samples obtained from children with OM, juvenile chinchillas were challenged with RSV followed 24 hours later by *M. catarrhalis* that had been admixed with NTHI. This regime, too, failed to produce the desired robust signs of experimental OM in the majority of animals. To overcome the possibility of failure due to competition between the two bacterial species when inoculated concurrently, as has been described for other bacterial species which share the same niche [25], [53], we established separate time points for the intranasal inoculation of animals with each bacterial agent. Thus, juvenile chinchillas were inoculated first with NTHI, followed four days later with *M. catarrhalis* and after an additional two days, RSV. This modified challenge regimen was intended to allow for first NTHI, then for *M. catarrhalis* to establish colonization of the NP prior to viral infection. As hoped, this latter multi-challenge regime resulted in exhibition of hallmark signs of OM in chinchillas, such as inflammation of the tympanic membrane [54], as well as several unique observations such as bullous myringitis and the presence of blood at the bullar orifice of the ET as observed upon dissection. These latter signs have not been noted in either the chinchilla model of adenovirus-predisposed OM due to NTHI [26] or when animals were challenged with either NTHI alone or with RSV alone [52] which suggested their unique association with *M. catarrhalis*-induced OM. Use of this model will allow for innovative *in vivo* studies designed to elucidate the molecular mechanisms of *M. catarrhalis* pathogenesis, as well as to aid in the assessment of vaccine candidates that target *M. catarrhalis.*

## Materials and Methods

### Ethics Statement

This study was performed in strict accordance with the recommendations in the Guide for the Care and Use of Laboratory Animals of the National Institutes of Health. The protocol was approved the Institutional Animal Care and Use Committee at The Research Institute at Nationwide Children’s Hospital (Protocol 01304AR, approved 9/27/10).

### Strains

*M. catarrhalis* strain 7169 was obtained via tympanocentesis from the middle ear of a child who had OM [55] and has been demonstrated experimentally to colonize the chinchilla nasopharynx [21]. RSV A2 was a generous gift from Dr. Mark E. Peeples, Nationwide Children’s Hospital, Columbus, OH, who purchased the virus from American Type Culture Collection. NTHI strain 86-028NP was isolated in 1986 [56] from the nasopharynx of a child undergoing tympanostomy and tube insertion for chronic OM at Nationwide Children’s Hospital, Columbus, OH. No further identifying patient information was obtained. This strain has been characterized and extensively used in chinchilla models of OM and a rat model of pulmonary clearance [22], [24], [27], [28], [29].

### Animals

As a proof-of-principle study, we first examined the ability of RSV to predispose to ascending *M. catarrhalis*-induced OM in a chinchilla host. To do so, six juvenile chinchillas (*Chinchilla lanigera*; Rauscher’s Chinchilla Ranch, LaRue, OH; mean mass 440±20 g) were challenged intranasally with 10^8^ CFU *M.* *catarrhalis* delivered in 0.2 ml sterile pyrogen-free saline (divided equally between the nares) to establish nasopharyngeal colonization. Two days later, chinchillas received 10^8^ PFU RSV delivered in 0.2 ml sterile pyrogen-free saline, divided equally between the nares (Table 1). Juvenile chinchillas have been previously demonstrated to be permissive to infection with RSV A2 [44], [52].

**Table 1:** Challenge protocol time table.

Day	Proof of PrincipleProtocol	Expanded Protocol
**−4**		NTHI 86-028 NP challenge
**0**	*M. catarrhalis* 7169challenge	*M. catarrhalis* 7169 challenge
**2**	RSV A2 challenge	RSV A2 challenge
**7**	Sacrifice (n = 3)	Sacrifice (n = 8)
**11**	Sacrifice (n = 3)	Sacrifice (n = 8)
**14**		Sacrifice (n = 8)
**17**		Sacrifice (n = 5)

Protocols to assess the ability of *M. catarrhalis* to induce OM in RSV-compromised juvenile chinchillas. For both protocols, nasopharyngeal mucosae, Eustachian tubes and middle ear mucosae were collected for culture at the time of sacrifice. Bullar washes were performed upon bisection of the bullae to recover planktonic *M. catarrhalis*.

Based on results obtained in our proof-of-principle study, as well as epidemiological data that suggests that *M. catarrhalis* requires a bacterial co-pathogen to induce robust disease, we next performed a larger cohort study designed to determine the ability of RSV to predispose to *M. catarrhalis*-induced OM when the chinchilla nasopharynx was co-colonized with both *M. catarrhalis* and NTHI. Toward this end, twenty-nine juvenile chinchillas (mean mass 352±43 g) were challenged intranasally with 10^8^ CFU NTHI then four days later with 10^8^ CFU.

*M. catarrhalis* to establish co-colonization in the nasopharynx. Two days later, animals were challenged intranasally with 10^8^ PFU RSV strain A2 (Table 1).

In both studies, chinchillas were monitored daily via video otoscopy (MedRx, Largo, FL) and tympanometry for signs of OM, such as erythema or vasodilatation of the tympanic membrane (TM), evidence of middle ear fluid behind the tympanic membrane or alteration in middle ear pressure outside the normal range for a healthy chinchilla (−100 to 150 daPa) [27], [30], [48]. Upon sacrifice, chinchillas were further classified based on gross pathological changes observed in the middle ear such as vasodilatation, erythema, hemorrhagic foci, bullous myringitis, the presence of fluid, biofilm formation and/or frank bleeding at the bullar orifice of the ET. Each middle ear was considered independent in these studies, and the percentage of middle ears displaying signs of OM was calculated.

### Sample Collection

Animals were sacrificed 7 and 11 days (both protocols), as well as 14 and 17 days (expanded protocol only) after challenge with *M. catarrhalis.* All bullae were bisected and images captured with VETDOC software (MedRx) and a Zeiss stereoscope (Stemi 200-C, New York City, NY). Bullae were washed with 100 µl saline and the recovered lavage fluids were serially diluted and plated onto chocolate agar to semi-quantitate the concentration of CFU for *M. catarrhalis* and NTHI. The left sagittal half of the skull and its associated tissues from the first chinchilla sacrificed were fixed in 10% neutral buffered formalin (Fisher, Pittsburgh, PA) for subsequent histological analysis. From the right sagittal half of the skull, mucosae from the NP, ET and middle ears were collected, weighed, suspended in 1 ml sterile pyrogen-free saline and homogenized with a Polytronic PT 1300D homogenizer (Kinematica, Bohemia, NY). Homogenized tissues were serially diluted and plated onto chocolate agar that contained 10 µg vancomycin/ml and 5 µg trimethoprim/ml (Sigma Aldrich, St. Louis, MO) to determine CFU *M. catarrhalis*/mg mucosa, and where necessary, on chocolate agar that contained 15 µg ampicillin/ml to determine CFU NTHI. Identification of *M. catarrhalis* was confirmed by Gram stain and Catarrhalis Disk Test (Fisher, Pittsburgh, PA).

### Scanning Electron Microscopy of Chinchilla Middle Ear Mucosae

Regions of middle ear mucosa proximal to the Eustachian tube opening were excised from the inferior bullae of the chinchilla middle ear. These mucosae were fixed with 2.5% glutaraldehyde (Electron Microscopy Sciences, Hatfield, PA) in 0.1 M phosphate buffer, pH 7.4, at 4°C overnight. Samples were post-fixed twice in 1% osmium tetroxide solution (Electron Microscopy Sciences) for 2 hr each, followed by five washes in distilled water. Samples were then dehydrated through a graded series of alcohol and dried with hexamethyldisilazane (HMDS; Electron Microscopy Sciences) for 10 min each. HMDS was removed and fresh HMDS was added before samples were allowed to air dry and a colloidal silver coat (Electron Microscopy Sciences) was used to adhere samples to Hitachi S-450 aluminum stubs (Electron Microscopy Sciences). Stubs were air dried overnight, then sputter coated with gold and palladium using a Cressington model 108 sputter coater (Watford, UK) prior to being viewed on a Hitachi S-4800 scanning electron microscope (Schaumburg, IL).

### Immunohistochemical Analysis of the Chinchilla Eustachian Tube

Five micron sections of paraffin-embedded ET from samples collected from the sacrificed chinchillas were deparaffinized and rehydrated through a graded series of alcohol. Slides were then blocked in 3% hydrogen peroxide for 15 min, washed in distilled water, then wash buffer (0.5 M Tris buffer with 0.05% Tween 20, pH 7.4). Superblock (Dako, Glostrup, Denmark) was applied for 10 min, followed by 10 min in wash buffer and 30 min in avidin/biotin block (Vector Laboratories, Inc., Burlingame, CA). Sections were then incubated with rabbit anti-*M. catarrhalis* whole cell lysate (WCL; a gift from Dr. Timothy Murphy, SUNY, Buffalo) diluted 1∶200 in wash buffer at 4°C, overnight. The slides were washed and blocked again with 3% hydrogen peroxide, followed by incubation with UltraTek anti-polyvalent solution (ScyTek, Logan, UT) for 30 min. *M. catarrhalis* detection was accomplished by means of a streptavidin-horseradish peroxidase complex and aminoethyl carbazole as a chromagen (ScyTek).

### Immunofluorescent Labeling of NTHI and *M. catarrhalis*

A biofilm retrieved from the middle ear of a chinchilla that had been challenged with NTHI, *M. catarrhalis* and RSV was embedded in OCT medium (Fisher, Pittsburgh, PA), then snap frozen on dry ice. Ten micron sections were immunolabeled to detect NTHI and *M. catarrhalis* within the biofilm using a standard protocol [31]. Briefly, the slide was air dried, fixed in cold acetone, then equilibrated in wash buffer (0.5 M Tris buffer with 0.05% Tween 20, pH 7.4). Sections were blocked with image-iT FX Signal Enhancer (Molecular Probes, Eugene, OR) as per the manufacturer’s instructions. The slide was incubated with polyclonal rabbit anti-NTHI outer membrane protein P5 [57] and, because of its role in biofilm formation [21], mouse anti-*M.* *catarrhalis* PilA (the major pilin subunit of the Type IV pilus of *M.* *catarrhalis*; a gift from Dr. Anthony Campagnari, SUNY Buffalo) [58] at 4°C overnight. The slide was then rinsed with wash buffer before incubation for 30 min with secondary antibodies (goat anti-rabbit IgG conjugated to AlexaFluor 488 and goat anti-mouse IgG conjugated to AlexaFluor 594; Invitrogen, Grand Island, NY). The sample was washed and mounted with ProLong Gold anti-fade reagent with 4,6-diamidino-2-phenylindole (DAPI) (Molecular Probes, Eugene, OR). Coverslips were placed over the sample and sealed. Sections were viewed using a Zeiss Axiovert 200 M inverted microscope (Zeiss, New York City, NY).

## Results

### Proof of Concept Study: Signs of OM were Present in Animals Challenged with *M. catarrhalis* and RSV

**Figure 1:**
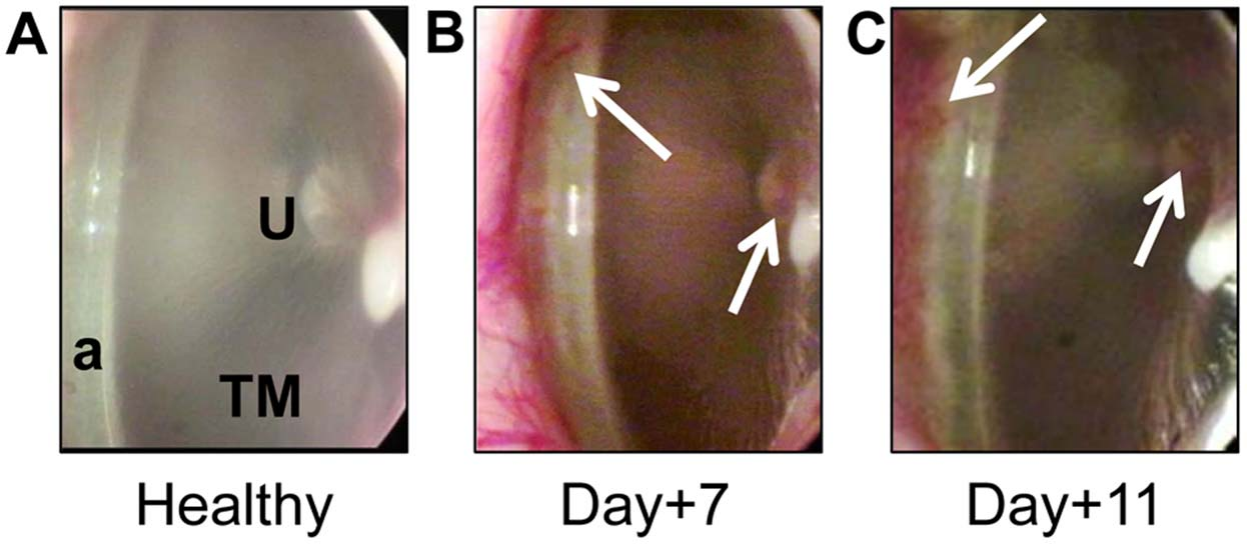
Clinically-relevant assessment for the development of experimental OM as determined by video otoscopy and tympanometry. Proof of concept study: (A) A healthy chinchilla tympanic membrane (TM), with both the annulum (a) and the umbo of the malleus (U) labeled. Panels B&C, chinchilla TM imaged seven or eleven days after *M. catarrhalis* challenge, respectively, with erythema (arrows) along the annulum and over the umbo of the malleus at its point of adherence to the TM.

To monitor chinchillas for the progression of OM, middle ear pressure was recorded via tympanometry, and we observed tympanic membranes daily via video otoscopy. The mean middle ear pressure (MEP) remained within the normal range for all chinchillas throughout the course of the study, with values that ranged from 17 to 45 daPa. Compared to their state prior to challenge, slight erythema along the annulum and the umbo of the malleus was observed in 6 of 12 ears (50%) 7 days after *M. catarrhalis* challenge and in 2 of 6 ears (33%) 11 days after *M. catarrhalis* challenge (Figure 1B and C, erythema is indicated by arrows). These signs, as observed via video otoscopy, are indicative of OM [27], [28], [32].

**Figure 2:**
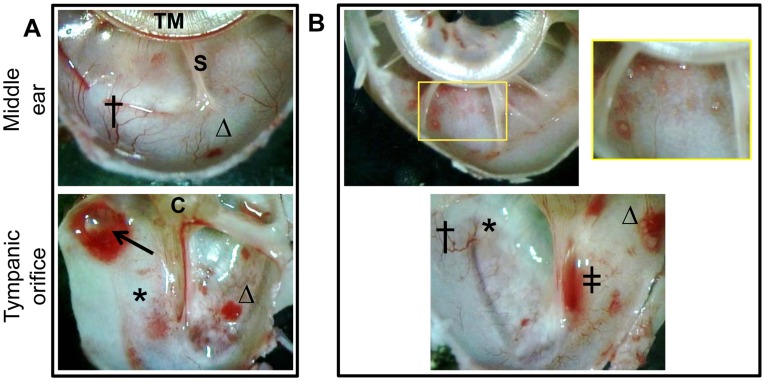
*M. catarrhalis*-induced inflammation within the chinchilla middle ear. Proof of concept study: Intranasal challenge of chinchillas with *M. catarrhalis* and RSV resulted in inflammation within the middle ear. (A) Seven days after bacterial challenge vasodilatation (†) was evident, as was focal submucosal edema (arrow) and erythema (*). Hemorrhagic foci (Δ) were present in the inferior bulla as well as near the opening of the Eustachian tube. (B) Eleven days after bacterial challenge bullous myringitis (box and inset) developed in addition to vasodilatation (†), erythema (*), hemorrhagic foci (Δ) and blood in the opening of the ET (‡). TM = tympanic membrane, S = septa, C = cochlea.

To visualize the inside of the middle ear, we sacrificed animals at the selected time points and bisected the inferior bullae. Upon dissection, vasodilatation was evident in the middle ear mucosa of all animals (Figure 2A, indicated by †). Grossly, upon bisection of the bullae, focal submucosal edema was observed in 4 of 12 ears (33%) (Figure 2B, indicated by an arrow). Submucosal hemorrhagic foci were present in the inferior bulla mucosa as well as near the opening of the ET in 10 of 12 ears (83%) (Figure 2A and B, indicated by Δ). A small volume of fluid (less than 5 µl) was retrievable from the middle ears of all animals; moreover, seven days after challenge with *M. catarrhalis*, one of three animals pre-designated for sacrifice at this time point had approximately 15 µl of culture-negative serosanguinous fluid present at the opening of the ET. Bullous myringitis was evident in one animal seven days after bacterial challenge and in two animals 11 days after bacterial challenge (Figure 2B, inset). One animal exhibited evidence of frank bleeding unilaterally in the bullar opening of the ET (Figure 2B, indicated by ‡).

### Proof of Concept Study: *M. catarrhalis* was Detected within Homogenates of Middle Ear Mucosae and Bullar Washes of Animals Challenged with *M. catarrhalis* and RSV

**Figure 3:**
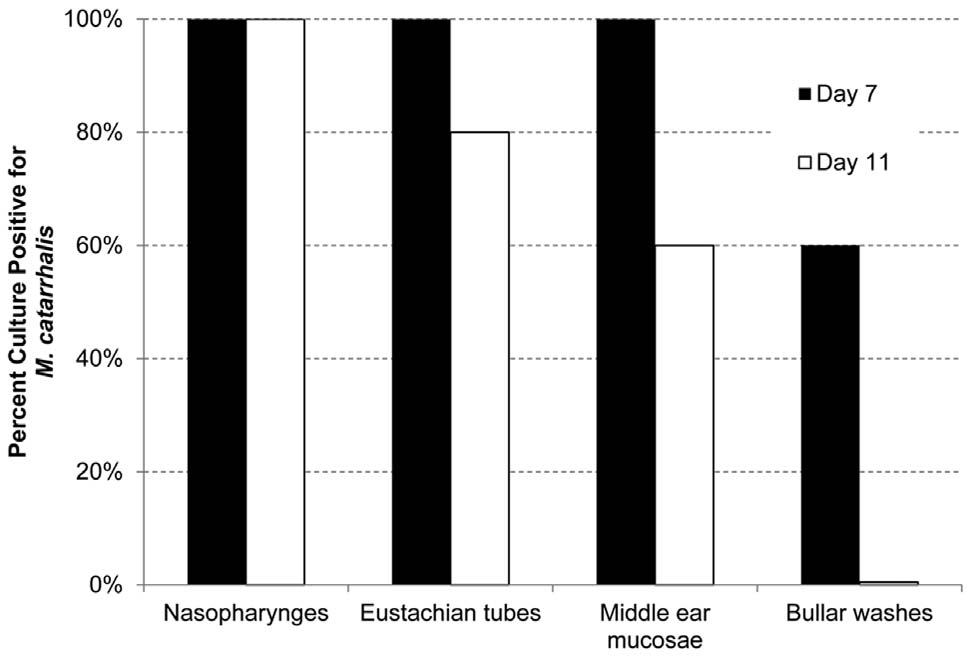
*M. catarrhalis* ascended from the chinchilla nasopharynx to the middle ear. Proof of concept study: *M. catarrhalis* was cultured from 100% of homogenates of nasopharynges, Eustachian tubes and middle ear mucosae seven days after exclusively intranasal bacterial challenge (white bars). In addition, 60% of ears remained culture positive for *M. catarrhalis* eleven days after intranasal bacterial challenge (black bars).

Seven days after bacterial challenge, 100% of homogenates (3 of 3 NP, 5 of 5 ET and 5 of 5 middle ear mucosae) and 60% (3 of 5 ears, 2 of 3 animals) of bullar washes were culture-positive for *M. catarrhalis* (Figure 3, black bars), with counts reaching 40 CFU/ml sample. A greater number of *M. catarrhalis* were detected in the NP (up to 1.1×10^3^ CFU/ml sample) than the ET (up to 3.2×10^2^ CFU/ml sample) or middle ear mucosae (up to 6.5×10^1^ CFU/ml sample). Eleven days after *M. catarrhalis* challenge, bullar washes were culture-negative for *M. catarrhalis*; however, 100% of nasopharyngeal homogenates (3 of 3 animals), 80% of ET homogenates (4 of 5 ears) and 60% of middle ear mucosae homogenates (3 of 5 ears) were culture-positive for *M.* *catarrhalis* (Figure 3, white bars). The number of *M. catarrhalis* present in the NP and ET remained constant, at approximately 1×10^3^ or 3.3×10^2^ CFU/ml, respectively. Of note, the concentration of *M. catarrhalis* detected within the middle ear nearly tripled between day 7 and 11, reaching 1×10^3^ CFU/ml. Therefore, we have provided culture data to show that *M. catarrhalis* was able to ascend the ET, adhere to and survive in the chinchilla middle ear for at least 11 days after intranasal challenge when partnered with RSV.

**Figure 4:**
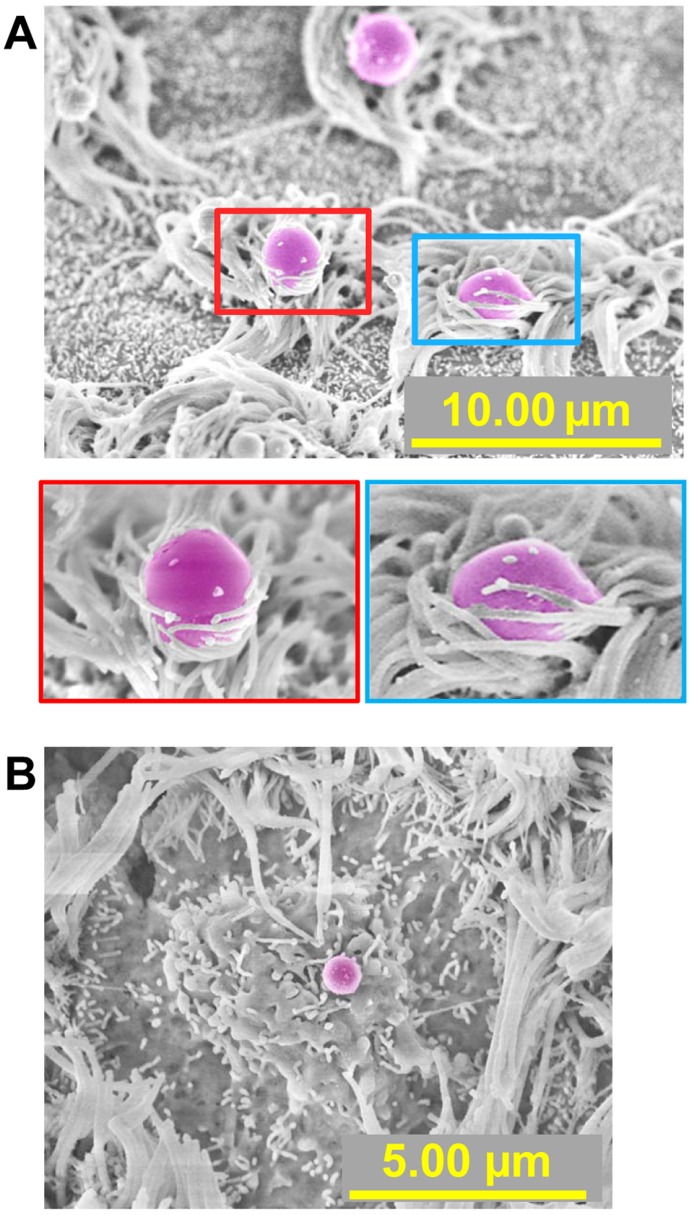
Detection of *M. catarrhalis* in the chinchilla middle ear. Proof of concept study: Scanning electron microscopy (SEM) images of chinchilla middle ear mucosa 11 days after intranasal *M. catarrhalis* challenge. *M. catarrhalis* (pseudo-colored purple) was detected on the mucosal surface in association with (A) cilia and also (B) with an elevated area of membrane that was ruffled in appearance.

To examine the physiological changes to host epithelial cells in the middle ear after challenge with *M. catarrhalis*, SEM was performed on a sample of chinchilla middle ear mucosa collected 11 days after *M. catarrhalis* challenge. Examination via SEM demonstrated the presence of bacteria with morphology typical of *M. catarrhalis* in tight association with ciliated cells (Figure 4A), as well as with an elevated area of ruffled epithelial cell membrane (Figure 4B). To the best of our knowledge, this is the first *in vivo* demonstration of *M. catarrhalis* in association with ciliated cells within the mammalian middle ear.

### Expanded Study: Clinically Relevant Signs of OM in Chinchillas Challenged with *M. catarrhalis*, NTHI and RSV

**Figure 5:**
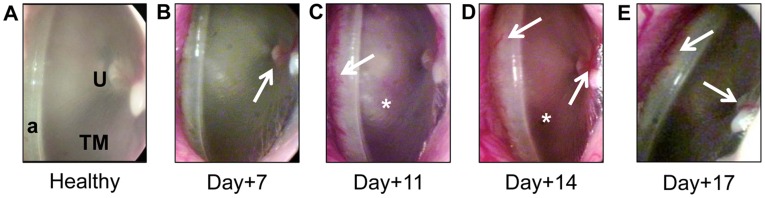
Clinically-relevant assessment for the development of experimental OM as determined by video otoscopy and tympanometry. Expanded study: Intranasal challenge of chinchillas with NTHI, *M. catarrhalis* and RSV resulted in clinical indicators of otitis media as determined by otoscopy and tympanometry. (A) A healthy chinchilla tympanic membrane (TM), with both the annulum (a) and the umbo of the malleus (U) labeled. (B) Seven days after *M. catarrhalis* challenge, erythema (arrow) was observed along the umbo, along with darkening of the TM. Panels C-E – TM 11, 14 and 17 days after *M. catarrhalis* challenge, respectively, erythema (arrows) was observed along the annulum, over the U and across the TM (*).

To monitor chinchillas for the progression of OM, middle ear pressure was recorded via tympanometry, and we observed tympanic membranes daily via video otoscopy. Whereas mean MEP remained within the normal range for all chinchillas throughout the course of the study, with values that ranged from 12 to 55 daPa; signs of inflammation were nonetheless observed. Compared to a healthy tympanic membrane (Figure 5A), erythema was observed along the annulum and the umbo of the malleus in 25 of 56 ears (45%) seven days after *M. catarrhalis* challenge (Figure 5B); in 26 of 42 ears (62%) eleven days after *M. catarrhalis* challenge (Figure 5C); in 13 of 26 ears (50%) fourteen days after *M. catarrhalis* challenge (Figure 5D); and in 5 of 10 ears (50%) seventeen days after *M. catarrhalis* challenge (Figure 5E). Fourteen days after *M. catarrhalis* challenge, erythema was also observed across the tympanic membrane (Figure 5D), indicative of greater disease severity when NTHI was included as a bacterial co-pathogen than that achieved in our proof-of-concept study wherein only *M. catarrhalis* and RSV were delivered via intranasal challenge.

**Figure 6:**
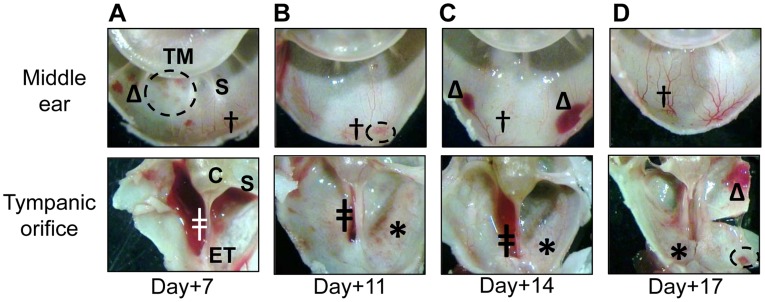
Gross morphological changes within the chinchilla middle ear after co-challenge with NTHI and *M. catarrhalis*. Expanded study: Chinchillas challenged intranasally with NTHI, *M. catarrhalis* and RSV demonstrated changes in the middle ear mucosa. Panels A-D: Seven, eleven, fourteen and seventeen days after challenge with *M. catarrhalis*, respectively, vasodilatation (†) and erythema (*) were evident. Bullous myringitis (encircled) was evident at days 7, 11 and 17 (panels A, B and D), and hemorrhagic foci (Δ) were present at day 14 and 17 (panels C and D). Moreover, 4 out of 16 animals (25%) exhibited blood (‡) in the opening of the Eustachian tube on days 11 and 14 (panels B and C). TM = tympanic membrane, S = septa, C = cochlea, ET = Eustachian tube.

In addition to vasodilatation and erythema within the middle ear mucosa, 8 of 58 middle ears (14%) developed bullous myringitis (Figure 6A, encircled). Focal points of sub-mucosal hemorrhage were also observed in a total of 46 of 58 ears (79%) throughout the study period (Figure 6C-D, indicated by Δ). Moreover, 4 of 16 animals exhibited unilateral frank bleeding at the opening of the ET on days 11 and 14 (Figure 6, indicated by ‡). All animals exhibited a limited volume of middle ear fluid (<5 µl) in at least one ear and a grossly visible (‘macro’) biofilm was retrieved from 1 of 16 ears eleven days after *M. catarrhalis* challenge, all of which indicated a greater severity of disease than was observed in our proof-of concept-study.

### Expanded Study: *M. catarrhalis* was Detected within Homogenates of Middle Ear Mucosa and Bullar Washes of Animals Challenged with *M. catarrhalis,* NTHI and RSV

**Figure 7:**
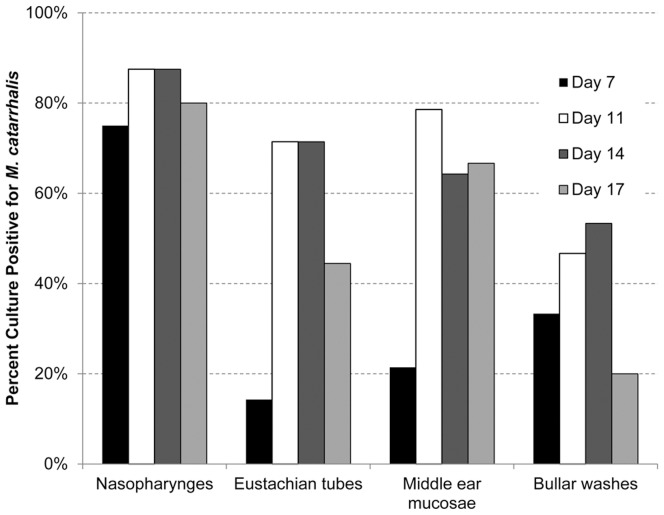
*M. catarrhalis* was detected adherent to multiple mucosal tissues. Expanded study: Percentage of mucosal sites that were culture-positive for *M. catarrhalis. M. catarrhalis* was detected in the middle ear as early as seven days and up to 17 days after intranasal challenge with *M. catarrhalis*.

To begin to establish the kinetics of *M. catarrhalis*-associated OM in the chinchilla model, homogenized NP, ET and middle ear mucosae were plated for recovery of this bacterium. *M. catarrhalis* was detected in the middle ear as early as seven days and for up to 17 days after intranasal *M. catarrhalis* challenge (Figure 7). The number of culture-positive NP, ET and middle ear mucosae increased from 7 to 11 days after challenge with *M. catarrhalis*, with a maximum of 3×10^2^, 8×10^2^ or 2×10^3^ CFU/ml homogenized sample recovered at this time point, respectively. Throughout the duration of the study, between 75 and 88% of samples recovered from the NP were culture-positive (e.g. up to 7 of 8 animals and yielding 2.5×10^4^ CFU/ml homogenized tissue), whereas with regard to samples from ET over the course of the study, 14 to 71% of samples were culture-positive (e.g. up to 10 of 14 ears and 7×10^3^ CFU/ml homogenized sample). Similarly, a maximum of 79% (11 of 14 ears) of middle ear mucosae and 53% of bullar washes (8 of 15 ears) were culture-positive for *M. catarrhalis* over the 17 days of the studied disease course (Figure 7), with the number of recoverable *M. catarrhalis* reaching 2×10^3^ or 1.6×10^3^ CFU/ml, respectively.

**Figure 8:**
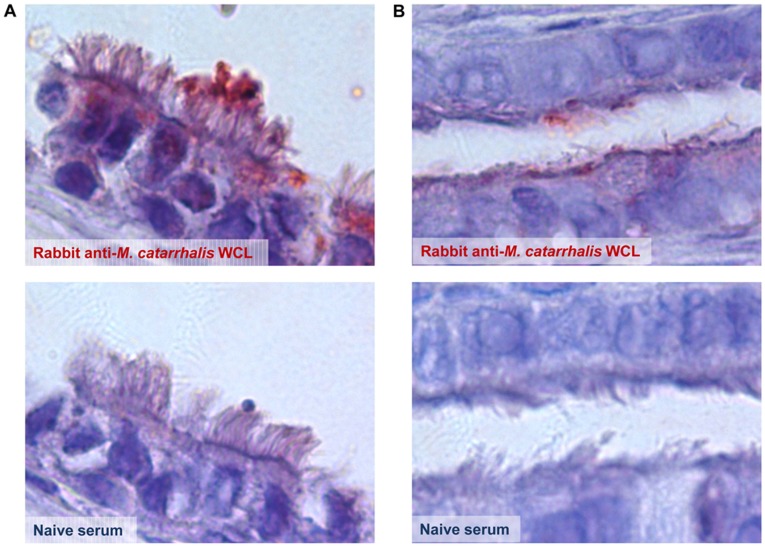
Detection of *M. catarrhalis* in the chinchilla middle ear. Immunohistochemistry of a chinchilla ET 11 days after challenge with *M. catarrhalis*. *M. catarrhalis* (red) was found associated with ciliated cells in both A) the middle ear and B) the ET. Images collected at100X. Rabbit anti-*M. catarrhalis* WCL = whole cell lysate.

In order to better characterize the relationship between *M.* *catarrhalis* and host epithelial cells, a cross section of the ET from an animal sacrificed 11 days after challenge with *M.* *catarrhalis* was labeled for the presence of this microbe using antibody directed against whole cell lysates of *M. catarrhalis*. Positive labeling both in the bullar opening of the ET (Figure 8A) and midpoint within the ET itself (Figure 8B) demonstrated that *M. catarrhalis* was present within the ET and middle ear of the chinchilla and appeared to be associated strictly with ciliated cells. This finding could prove to be pertinent to our evolving understanding of *M. catarrhalis* pathogenesis.

**Figure 9:**
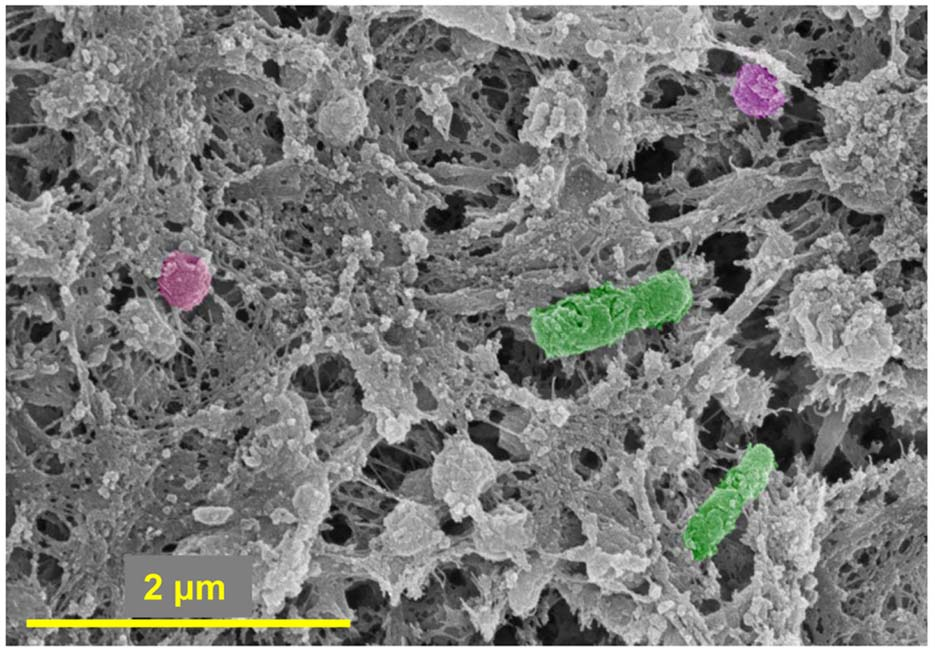
Detection of *M. catarrhalis* and NTHI in biofilms recovered from the chinchilla middle ear. A biofilm recovered from the middle ear that contained NTHI (pseudo-colored green) and *M. catarrhalis* (pseudo-colored magenta). Imaged by scanning electron microscopy.

**Figure 10:**
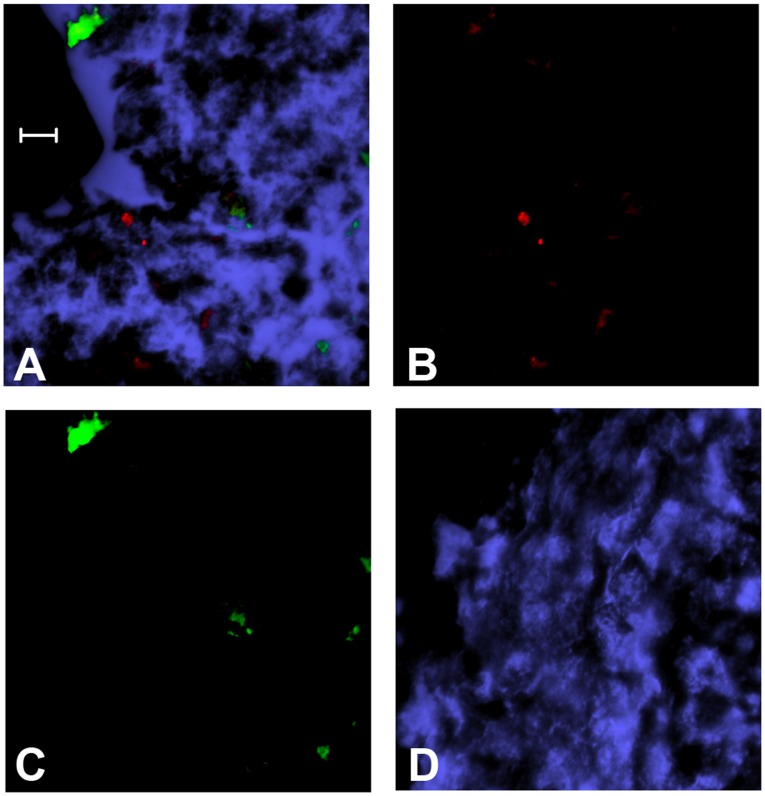
Detection of *M. catarrhalis* and NTHI in biofilms recovered from the chinchilla middle ear. Immunofluorescence image of a biofilm incubated with rabbit-anti NTHI outer membrane protein P5 (green), mouse anti-*M. catarrhalis* PilA (red), and stained with DAPI to label DNA (blue). A) Merged image, B) *M. catarrhalis*, C) NTHI, D) serial section control demonstrating no labeling of either *M. catarrhalis* or NTHI. Scale bar = 5 µm.

To characterize the bullous myringitis that developed in 11 total animals, a sample of mucosa that contained a bullous myringitis blister was processed and evaluated via SEM. This specimen was subsequently determined to be associated with a polymicrobial micro-biofilm (Figure 9). Bacteria within this biofilm were preliminarily identified as *M. catarrhalis* and NTHI based on bacterial size, morphology and culture. Additionally however, the macro biofilm from a chinchilla that was sacrificed 11 days after *M. catarrhalis* challenge was sectioned and dual-labeled with antibodies directed against either *M. catarrhalis* or NTHI to confirm the presence of these organisms. Positive labeling of both species of bacteria can be seen in Figure 10. Thus, we determined that upon co-challenge with RSV, both *M. catarrhalis* and NTHI ascended from a colonization site in the NP into the middle ear, where they formed both micro and macro biofilms. Based on these data, we hypothesized that the formation of mixed polymicrobial biofilms, both micro and macro, may be important in pathogenesis of *M. catarrhalis*-induced OM.

## Discussion

In children, viral infection predisposes to bacterial OM by facilitating ascension of select members of the colonizing NP flora into the middle ear space. Thereby, in order to best mimic this natural course of disease, it is logical that experimental models of OM incorporate viral predisposition. It is also shown that an appropriate partnering of virus and bacterium is required, as dysregulation of ciliary function, altered mucus production by the respiratory epithelium and reduction in secretion of host defense molecules may be altered in a virus-specific manner. For example, infection of chinchillas with adenovirus serotype 1 is shown to result in thickened mucus overlaying the respiratory mucosa, which is favored by NTHI [26], [59], [60], whereas influenza A virus induces the production of thinner secretions, which is optimal for *S. pneumoniae* [61], [62]. The timing and order of viral-bacterial challenge is also critical. In the adenovirus-NTHI superinfection model, virus is administered seven days prior to bacterial challenge and results in the development of experimental OM in 88–100% of chinchillas [24], [27]. In contrast, to induce pneumococcal OM, *S.* *pneumoniae* is administered first; followed two days later by influenza A virus and results in 67% of chinchillas that develop experimental OM [61], [62].

Prior work demonstrated that adenovirus serotype 1 is not an appropriate partner for *M. catarrhalis* [48]. Accordingly, and with epidemiologic data to suggest a critical role for RSV in the incidence of OM, we adapted our challenge strategy. Thereby, in our initial attempts to develop a model of ascending *M. catarrhalis*-induced OM, chinchillas were challenged intranasally with RSV followed by *M. catarrhalis* or challenged intranasally with RSV, then a mixed inoculum of *M. catarrhalis* and NTHI. In both experiments, a maximum of 50% of middle ear mucosae (2/4 and 3/6, respectively) were culture-positive for *M. catarrhalis* (unpublished data). Nonetheless, and as in the studies described herein, vasodilatation and hemorrhagic foci were observed in the middle ear space of chinchillas upon sacrifice, which demonstrated what might have been early predictive hallmark signs of *M.* *catarrhalis* ascension into the chinchilla middle ear. Based on these preliminary experimental data and epidemiological evidence, we herein attempted to optimize the model further by establishment of bacterial colonization before viral challenge in order to facilitate more robust ascension of *M. catarrhalis* from the NP into the middle ear space. This experimental model bears physiological resemblance to the disease process in children with OM, given that nasopharyngeal flora ascend into the middle ear space once viral infection has compromised ET function and created a more permissive environment for bacterial infection [11], [17], [44], [52].

Here, intranasal challenge with *M. catarrhalis* followed by RSV resulted in subtle, though likely ‘clinically’ relevant signs of OM as determined via video otoscopy, despite the fact that the mean middle ear pressure remained within the normal range for the chinchilla. At sacrifice, multiple signs of inflammation were observed in the middle ear mucosa, such as vasodilatation, submucosal edema, erythema and bullous myringitis. A volume of <5 µl of middle ear fluid was retrieved from most middle ears, and one middle ear contained approximately 15 µl of culture-negative serosanguinous fluid within the bullar opening of the ET. Signs of OM were evident via video otoscopy 4 days after bacterial challenge; moreover, the NP, ET and middle ear mucosal homogenates were culture-positive for *M. catarrhalis* within seven days after bacterial challenge, which indicated the ascension of *M. catarrhalis* from the nasopharynx into the middle ear space. The relative quantity of *M. catarrhalis* detected within the middle ear space was greater eleven days after intranasal challenge with *M. catarrhalis* than at seven days after challenge, indicating bacterial replication in the middle ear. Further, samples of the middle ear mucosae that were analyzed via scanning electron microscopy demonstrated *M. catarrhalis* in tight association with ciliated middle ear mucosal cells and with an elevated area of ruffled epithelial cell membrane. Previous studies have demonstrated an affinity of *M. catarrhalis* for ciliated cells *in vitro* [63], but to the best of our knowledge, this is the first *in vivo* demonstration of ciliotropism by *M. catarrhalis*. Therefore, although this model lacked the robustness of the signs or severity typically observed in animal models of OM upon challenge with NTHI or *S. pneumoniae*, such as profuse effusion, extensive formation of biofilm, labyrinthitis, *etc*., it nonetheless had potential for further development as well as utility for studies of *M. catarrhalis* pathogenesis. Further, this model mimicked the natural disease in children, wherein *M. catarrhalis* induces a less severe disease than do other bacterial species [37], [38], [51].

Epidemiological studies demonstrate that OM is a polymicrobial disease and that bacterial species, especially *M. catarrhalis,* are often cultured in conjunction with other bacteria [49], [64], [65]. Moreover, *M. catarrhalis* and NTHI have been shown to benefit each other *in vitro* by promoting adherence and biofilm formation [25], [66], [67]. Further, NTHI and *M. catarrhalis* are frequently co-cultured from the nasopharynges of children [49]. We hypothesized that co-colonization of the chinchilla nasopharynx by NTHI was essential for ascension of *M. catarrhalis* through the ET and into the middle ear based on previous work which demonstrated that co-challenge with RSV and *M. catarrhalis* alone failed to produce the desired robust infection (Bakaletz, unpublished data). In animals challenged with NTHI, *M. catarrhalis* and RSV, more severe clinical signs of disease were evident as observed by video otoscopy than had been observed in our more simple *M. catarrhalis*-RSV dual challenge proof-of-concept study; moreover, the number of recoverable *M. catarrhalis* from both the ET and middle ear were greater when chinchillas were intranasally challenged with NTHI, *M. catarrhalis* and RSV as compared to our proof-of-concept study. We are actively investigating the molecular mechanisms by which co-colonization of the NP with NTHI and *M. catarrhalis* promoted ascension and increased the persistence of *M. catarrhalis* in the chinchilla middle ear, while simultaneously repressing signs typically seen with NTHI-induced OM in the chinchilla [22], [24], [26], [28], [31]. Further, upon sacrifice in the expanded study, blood was observed in the bullar opening of the ET seven days after *M. catarrhalis* challenge and persisted for fourteen days after *M. catarrhalis* challenge in 25% of animals. The presence of frank bleeding was reminiscent of earlier reports of epistaxis in macaques challenged with *Moraxella* sp. [68], [69] and suggested that bleeding may be a hallmark sign of *Moraxella*-induced disease of the uppermost airway in animal models. Another potential hallmark sign of *M. catarrhalis*-induced OM was the formation of both macro and micro biofilms in the middle ear space of chinchillas challenged via this protocol. The presence and characteristics of polymicrobial biofilms, both micro and macro, in a model of *M. catarrhalis*-induced OM has not been evaluated previously; however, we believe this to be an important aspect of the model that warrants further investigation, given the overall importance of biofilms to the chronic and recurrent nature of OM [22], [32], [70]. Armbruster *et al*. have studied biofilms formed *in vitro* by *M. catarrhalis* and NTHI and have determined that the two species communicate via quorum sensing and that the presence of NTHI in an *M. catarrhalis* biofilm increases persistence of *M. catarrhalis* [25]. Regardless of the role of macro *versus* micro biofilms in experimental OM, the formation of biofilms and the data which demonstrated that the NP and middle ear were culture-positive for *M. catarrhalis* for the duration of the study indicated that *M. catarrhalis* was adherent to ciliated epithelial cells and had survived and multiplied within the chinchilla middle ear after ascending to that site under its own power.

In summary, the field of *M. catarrhalis*-induced OM research to date has been strongly hindered by the lack of an experimental model in which *M. catarrhalis* ascends from the NP into the middle ear to cause disease. Our work has provided support for epidemiological studies that have demonstrated the role of RSV predisposition to OM and the importance of mixed bacterial infections – specifically NTHI and *M. catarrhalis* – in OM. This model utilized a strictly ascension-based approach (as opposed to a transbullar challenge), which is highly physiologically relevant and has resulted in development of OM in the majority of chinchillas studied. The continued development of this animal model will facilitate a better understanding of *M. catarrhalis* pathogenesis in this polymicrobial disease. Additionally, this model has potential to provide a means to investigate strategies to prevent or treat OM due to *M. catarrhalis*.
